# Intraoperative visualization of cerebral aneurysms using navigated 3D-ultrasound power-Doppler angiography

**DOI:** 10.1007/s00701-024-06310-9

**Published:** 2024-10-19

**Authors:** Andrej Šteňo, Ján Buvala, Sofia Malchárková, Magdaléna Mižičková, Rastislav Bažík, Peter Mikula, Ivan Bízik, Juraj Šteňo

**Affiliations:** 1 Department of Neurosurgery, Faculty of Medicine of Comenius University and University Hospital Bratislava, Limbová 5, Bratislava, 833 05 Slovakia; 2 Department of Radiology, Faculty of Medicine of Comenius University and University Hospital Bratislava, Bratislava, Slovakia; 3 Clinical Neuroscience Research Unit, Department of Neurosurgery, Faculty of Medicine of Comenius University and University Hospital Bratislava, Bratislava, Slovakia

**Keywords:** Aneurysm, Clipping, Ultrasound, Power-Doppler, Navigation

## Abstract

**Background:**

The questions of whether the spatial resolution of navigated 3D-ultrasound (3D-US) power-Doppler angiography imaging rendered by existing 3D-US systems is sufficient for the intraoperative visualization of cerebral aneurysms, and in what percentage of cases, are largely unanswered. A study on this topic is lacking in the literature.

**Methods:**

From 2015 to 2022, we performed 86 surgeries on 83 aneurysm patients. Navigated 3D-US was used at the discretion of the operating neurosurgeons when available (i.e., not being used during parallel tumor surgeries). Twenty-five aneurysms (15 ruptured) were operated on using 3D-US; 22 aneurysms were located at the middle cerebral artery (MCA). Patient 3D-US power-Doppler angiography images and surgical reports were retrospectively reviewed to assess the intraoperative ultrasound visibility of aneurysms.

**Results:**

In 20 patients (80%) the aneurysms were successfully visualized. In five patients (20%), the aneurysms visualization was insufficient or absent. Nineteen of 22 aneurysms (86.4%) were visualized in the MCA aneurysm subgroup. We observed no association between aneurysm visibility and aneurysm size or the presence of subarachnoid hemorrhage. In the subgroup of MCA aneurysms, no association between aneurysm visibility and the presence of subarachnoid hemorrhage was found; a trend toward poor sonographic visibility of smaller aneurysms was observed (*p* = 0.09).

**Conclusions:**

Our initial data show that intraoperative 3D-US power-Doppler angiography, rendered by current navigated 3D-US systems, clearly depicts the majority of aneurysms in the MCA aneurysm subgroup. However, future prospective studies performed on a higher number of aneurysms localized at various anatomic sites are needed to confirm our initial findings and determine their potential clinical relevance.

## Introduction

 Navigated 3D-ultrasound (3D-US) power-Doppler (PD) mode angiography enables intraoperative visualization of cerebral vascular anatomy and localization of brain vessels in their actual position using pointer [68]. While visualization of normal cerebral vessels, including small-diameter perforating arteries, using navigated 3D-US PD angiography is relatively well-known [61, 62], data on the intraoperative visualization of cerebral aneurysms are scarce. Apart from an assessment of the imaging of five aneurysms in a retrospective study evaluating the sonographic visibility of 162 different brain lesions [40], a study on this topic is lacking in the literature.

Early and precise identification of the position of cerebral aneurysm neck and dome as well as localization of surrounding vasculature is an important step during aneurysm clipping. Despite this process often being uncomplicated (especially in unruptured cases), in certain situations, such as during surgeries for distal aneurysms or in the presence of large intracerebral/intrasylvian hematomas, massive brain swelling, or extensive scarifications, the identification may be relatively challenging [22, 28, 33, 43]. The creation of a “mental 3D image” [34] of the aneurysm and nearby vessels can be difficult in such situation. While conventional neuronavigation based on a variety of preoperative imaging modalities has been found to be helpful in facilitating surgeons’ 3D orientation and identifying the position of aneurysms in different anatomical locations (using a pointer or navigation-linked head-up displays) [8, 14, 23, 24, 35, 43, 46, 53, 57, 65], the gradually increasing shift of vascular structures can cause considerable navigation inaccuracy [35, 50, 53]. Several centers have described the use of various intraoperative imaging modalities that allow navigation data update and brain-shift compensation, such as computed tomography (CT) angiography [7, 66], intraoperative magnetic resonance imaging (MRI) angiography [39], or (navigable) intraoperative digital subtraction angiography (DSA) [11, 31, 44, 52]. However, a drawback of these modalities is high costs.

Navigated 3D-US PD angiography is surely a more cost-effective intraoperative imaging modality. However, several factors can negatively influence the quality of 3D-US PD mode visualization of aneurysms. First, 3D-US images are generally inferior compared to 2D-ultrasound (2D-US) images. This difference most likely reflects the quality of the software interpolation of missing data between 2D slices during the scans [2] and the fact that tremor during the freehand 3D-US image acquisition may be recognizable in the 3D-US volumes, leading to incorrect shape visualization [5]. Second, the quality of the 3D-US image rendering is strongly determined by the algorithm used for 3D-US reconstruction [56, 68]. Lastly, the quality of the 3D-US PD mode visualization of aneurysms is also determined by the quality of the 2D images, which can also be negatively influenced by several factors: the PD signal insonation angle dependency [32, 51]; the spinning turbulent blood flow that may cause the absence of PD signals in the center of the aneurysm [48], the decrease in cerebral blood flow and blood flow in the aneurysm sac as a result of subarachnoid hemorrhage (SAH) [20]; the PD motion sensitivity (flush) artifacts [47, 51]; the tendency of the PD signal to expand beyond the vessel walls (blooming), causing vascular structures to seem thicker than they really are [47]; and the overall lower resolution of PD compared to other imaging modalities [13, 42]. Hence, due to the absence of a study on the visualization of cerebral aneurysm, the quality of their imaging using navigated 3D-US PD angiography is uncertain. The questions of whether the spatial resolution of 3D PD angiography imaging rendered by existing navigated 3D-US systems is sufficient for imaging of cerebral aneurysms, and in what percentage of cases, are largely unanswered. Of note, unlike in intraoperative DSA, CT, and MRI angiography, data on aneurysm imaging using 3D-US angiography cannot be extrapolated from (hundreds of) publications on preoperative aneurysm imaging – as 3D-US PD angiography can only be performed intraoperatively, data regarding this modality are much rarer.

Considering the aforementioned facts and our positive experience with 3D-US vascular imaging during brain tumor surgeries [58–62], we decided to at least partially fill this gap of knowledge by providing a retrospective analysis of the ability of navigated intraoperative 3D-US PD angiography to visualize cerebral aneurysms in our series of aneurysm surgeries.

## Materials and methods

### Patient selection

The first surgery (a glioma resection) using navigated 3D-US was performed at our department in 2010. The first aneurysm clipping with utilization of 3D-US PD angiography was performed in January 2015. Between January 2015 and December 2022, 86 surgeries for cerebral aneurysm were performed on 83 patients in our department; 57 patients harbored aneurysm on the middle cerebral artery (MCA).

In a total of 25 surgeries performed on 25 patients (22 having an MCA aneurysm), navigated 3D-US using PD angiography was used; 15 out of 25 aneurysms were ruptured. During aneurysm clipping, 3D-US was used at the discretion of operating neurosurgeons and only in situations when it was not needed during parallel tumor operations (as there is a relatively large amount of published data indicating benefits of 3D-US utilization in brain tumor surgeries [18, 19, 61, 62, 68], as opposed to the general lack of data regarding its use during aneurysm clipping).

### Intraoperative 3D-US PD angiography utilization

A stand-alone navigated 3D-US system (SonoWand Invite, SONOWAND AS, Trondheim, Norway) was utilized from January 2015 to July 2019; from August 2019 to December 2022, a newer navigated 3D-US system was used (Ultrasound bk5000, BK Medical Aps, Herlev, Denmark integrated with neuronavigation Curve, Brainlab AG, Munich, Germany). Both systems enable automatic fusion of the intraoperative 3D-US PD data with preoperative CT or MRI navigation sequences and provide rendering of ultrasound images in standard three orthogonal planes. The preoperative navigation data were based on 3D CT angiography in 24 cases, and in one case on 3D MRI angiography.

Ultrasound scanning techniques used in our department were described in our previous reports [60–63]; the scanning during aneurysm surgery is almost identical. Briefly, ultrasound probes used during surgeries were equipped with a reflective marker reference frame. The 3D-US data were acquired by freehand probe movement, tracking the position of the given probe by means of the navigation camera. Duplex PD ultrasound, consisting of two components: brightness mode (B-mode) showing neural brain structures and hematomas, and PD mode showing blood flow (i.e., ultrasound angiography), was used during the scanning. To minimize the PD flush artifacts, which appear as red fields or stripes on the 3D-US image after PD detects motion of the saline [47] (e.g., within the Sylvian fissure after its partial opening), the freehand probe movements were performed significantly slower than during standard B-mode 3D-US scanning during tumor surgeries.

After scanning the operating field, 3D-US B-mode and angiography images rendered in standard axial, coronal, and sagittal planes and fused with preoperative navigation CT (or MRI) data were used for navigation. In cases where the operating neurosurgeons considered accurate navigation to be helpful (e.g., to enable exact localization of the aneurysm dome and neck in the case of a large shift caused by partial evacuation of a hematoma), the ultrasound data were updated. The data update usually took approximately 2–3 min. To confirm complete aneurysm occlusion and blood flow in surrounding vessels after clipping, standard indocyanine green video angiography (and not 3D-US angiography) was used.

### Study design

**Fig. 1:**
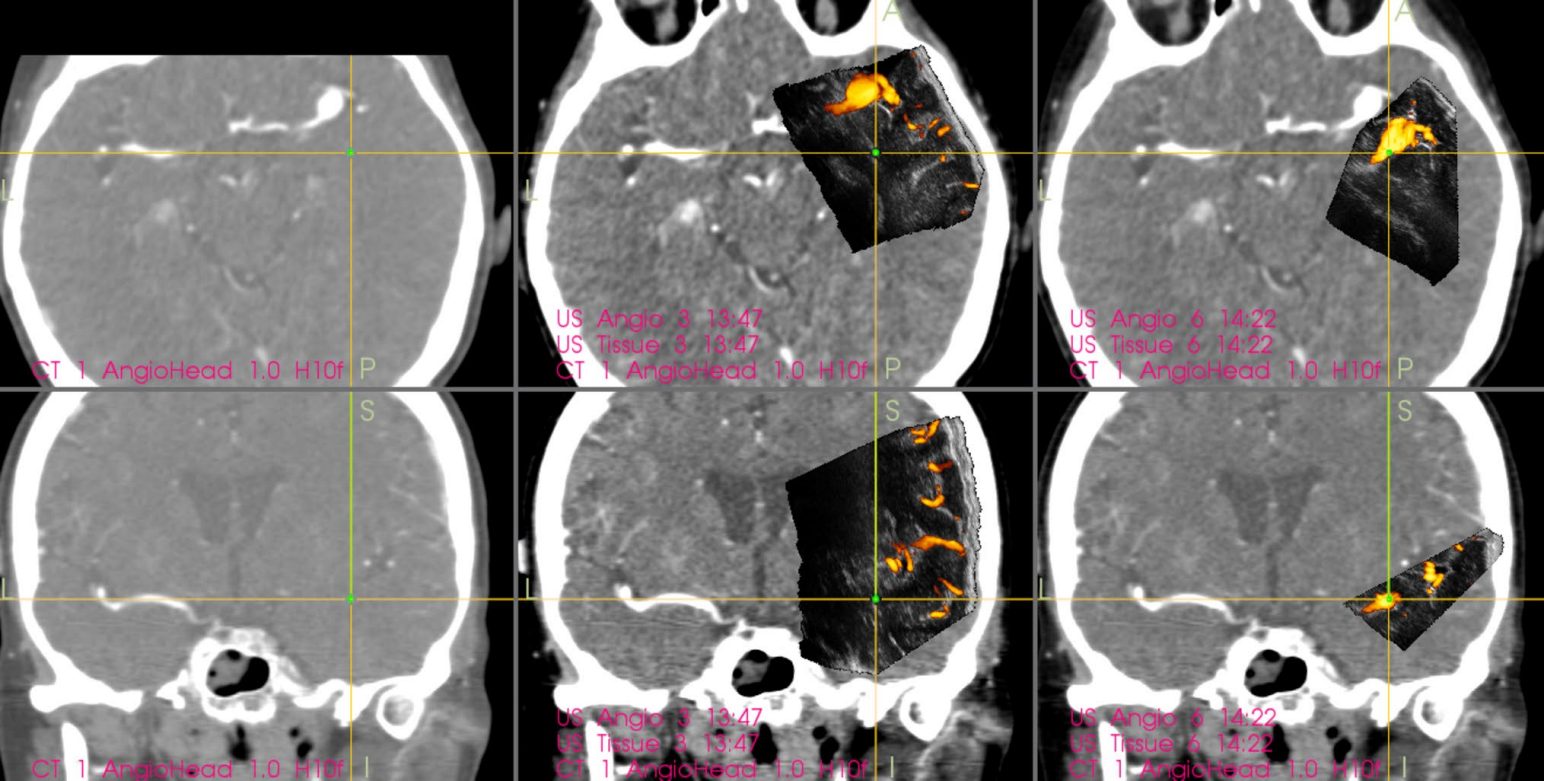
Example of an MCA aneurysm distinctly depicted by the navigated intraoperative 3D-US power-Doppler angiography. Left column: Preoperative navigation CT angiography showing an unruptured right-sided MCA aneurysm. (case No 18). Middle column: Initial 3D-US power-Doppler image acquired before the start of dissection; note the aneurysm is almost in the same location as depicted on preoperative CT angiography. Right column: Another 3D-US power-Doppler image acquired after partial dissection of the Sylvian fissure, note the significant shift of the aneurysm. Center of the yellow cross is located in the area of the aneurysm neck

Data from navigated 3D-US PD angiography images and patients’ operative reports were retrospectively reviewed. The aneurysm visibility evaluation was based on the study conducted by Policicchio et al. [40], who assessed ultrasonic visibility of various brain lesions, including five aneurysms; only distinctly depicted aneurysms were considered “visible” (Fig. 1).

The correlation between aneurysm visibility and aneurysm size, as well as the correlation between aneurysm visibility and the presence of SAH, were assessed. In cases where the aneurysms were not depicted by 3D-US PD angiography, the potential reasons for the lack of aneurysm visualization were assessed on a case-by-case basis reviewing patients’ operative reports and evaluating their imaging data (stored 3D-US PD angiography screenshots).

### Statistical methods

The patients’ data were summarized and analyzed using descriptive and inferential statistics. Categorical variables (aneurysm visibility and aneurysm rupture) are presented as counts and percentages in a contingency table. Fisher’s exact test was used to determine if there was a nonrandom association between the two variables. The size of the aneurysm as a continuous variable was first checked for normality using the Shapiro-Wilk test and subsequently tested for between-group differences using the Welch Two Sample t-test. All tests were two-tailed and performed at a significance level α = 0.05 using StatsDirect 3.3.6 software (StatsDirect Ltd., Cheshire, UK).

## Results

Clinical, demographic, radiological, and surgical data of 25 aneurysm patients operated on using navigated 3D-US angiography are summarized in Table 1.

**Table 1:** Clinical, demographic, radiological and surgical data of 25 aneurysm patients operated using navigated 3D-US PD angiography

Case No.	Age/sex	Aneurysm location	Maximal neck/dome dimension (mm)	Hunt-Hess grade	Type of aneurysm treatment	3D-US angiography depicted aneurysm distinctly	GOS score at hospital discharge
1.	51y/female	MCA	2,5/3,5	2	Clipping	Yes	5
2.	57y/female	MCA	3/10	2	Clipping	Yes	5
3.	68y/male	MCA	5/10	Unr	Clipping	Yes	5
4.	69y/female	MCA	3/7	Unr	Clipping	Yes	4
5.	56y/female	PICA	3/6	Unr	Clipping	No	5
6.	66y/female	MCA	2/5	Unr	Clipping	Yes	5
7.	44y/female	MCA	3,7/5	Unr	Clipping	Yes	5
8.	62y/female	MCA	4,5/5	2	Clipping	Yes	N/A*
9.	58y/female	MCA	2,5/6	Unr	Clipping	Yes	5
10.	67y/male	MCA	3,5/7	5	Clipping	Yes	1
11.	59y/female	MCA	2/5	5	Clipping	Yes	3
12.	60y/female	MCA	3/8,5	3	Clipping	Yes	5
13.	60y/female	MCA	2,8/6	Unr	Clipping	No	5
14.	73y/female	MCA	4/6	3	Clipping	Yes	3
15.	72y/male	MCA	4/6	Unr	Clipping	Yes	5
16.	58y/female	MCA	2/2	Unr	Clipping	Yes	5
17.	39y/female	MCA	3/8	5	Clipping	Yes	1
18.	47y/female	MCA	2,5/3,5	3	Clipping	No	5
19.	63y/female	MCA	4,5/13	Unr	Wrapping + ET	Yes	5
20.	44y/male	MCA	3/4,5	3	Clipping	No	3
21.	46y/male	Pcom	2/7,5	5	Clipping	Yes	2
22.	41y/male	Acom	6,5/19	5	Clipping	No	3
23.	47y/male	MCA	4/8	2	Clipping + ET on a delayed basis**	Yes	N/A***
24.	41y/female	MCA	3/5,5	5	Clipping	Yes	1
25.	69y/female	MCA	2/4,6	3	ET on a delayed basis (Fig. 7)	Yes	N/A***

*The patient, suffering early preoperative vasospasm and postoperative brain ischemia, was transferred to intensive care unit to a hospital at his place of residence five days after the clipping (despite early vasospasms, ET was declined by interventional radiologists); GOS could not be evaluated due intubation and sedation which confounded neurological status [72] **The ruptured part of aneurysm was located on its neck and only tangential clip application was possible; the patient was subsequently treated endovascularly on a delayed basis (emergent endovascular treatment was declined by interventional radiologists, as dual antiplatelet therapy was necessary) ***Both patients had to be transported intubated and sedated to a hospital providing ET (for technical reasons interventional radiology was not available in our hospital building that time); GOS could not be evaluated due intubation and sedation

Acom = Anterior Communicating Artery, ET = Endovascular Treatment, GOS = Glasgow outcome scale, MCA = Middle Cerebral Artery, mm = millimeters, N/A = not applicable, Pcom = Posterior Communicating Artery, PICA = Posterior Inferior Cerebellar Artery, Unr = unruptured aneuryms

### Sonographic visibility of aneurysms

**Fig. 2:**
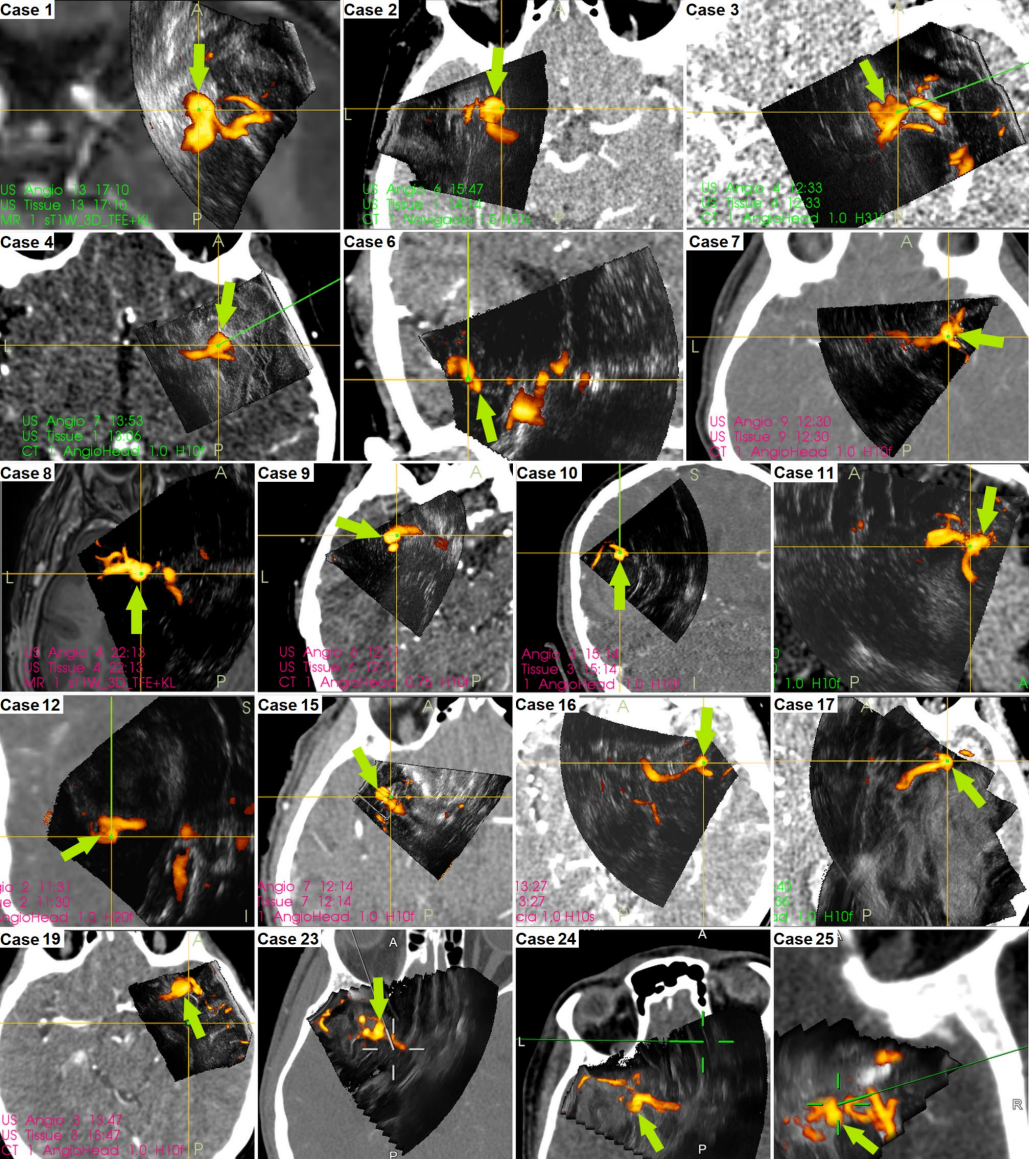
Overview of intraoperative 3D-US angiography images of 18 patients with distinctly depicted MCA aneurysms. Green arrows are pointing at the aneurysms. *In patient No. 14 no screenshots were available for technical reasons; however, the distinct aneurysm sonographic visibility could be reliably assessed based on the operating report

**Fig. 3:**
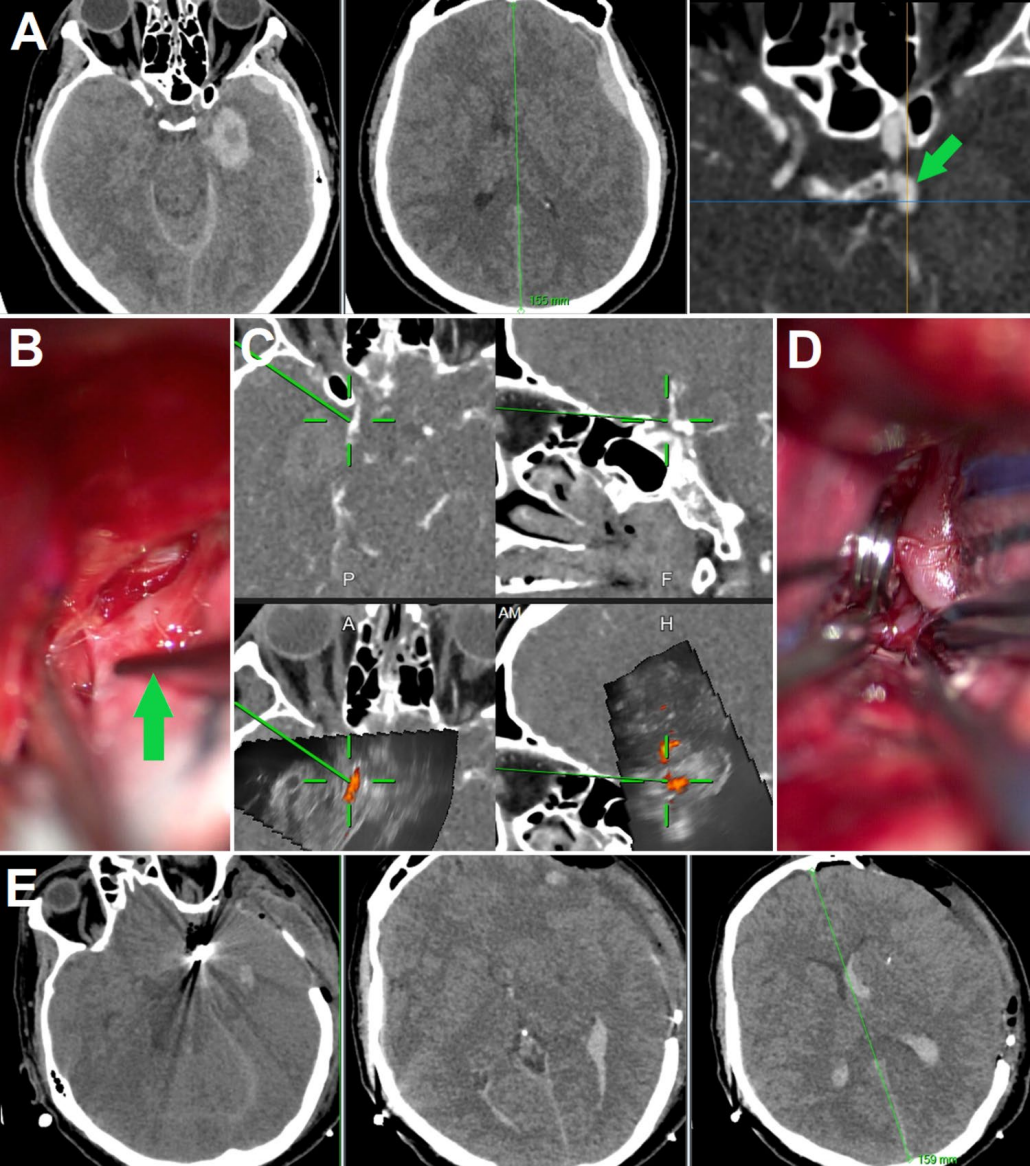
**Ruptured posterior communicating artery aneurysm (case No. 21) A:** CT and CT angiography images showing intracerebral and subdural hematoma after rupture of a left-sided Pcom aneurysm *(green arrow)*. **B:** Tip of the pointer *(green arrow) *is located in the presumed localization of aneurysm neck, note significant adhesions localized under the pointer tip complicating anatomical orientation. **C:** Confirmation of the aneurysm neck localization (*center of the green cross*) using updated 3D-US images, the aneurysm shift is minimal (as compared with preoperative CT angiography). Repeated visualization of the aneurysm dome heading into the temporal lobe subliminally “reminded” the surgeon not to exert any pressure on the temporal lobe. This would likely be unnecessary for an experienced cerebrovascular surgeon, but may be useful for surgeons who do not operate on similar cases routinely and may help prevent premature aneurysm rupture [27] **D:** Visual inspection after aneurysm clipping. **E:** **H** CT images performed on the first postoperative day. Note the absence of the midline shift (the bone-flap was not inserted and duraplasty was performed immediately after aneurysm clipping and hematoma evacuation)

In 20 patients (80%), the aneurysms were successfully visualized; in five patients (20%), the aneurysms were not depicted/not distinctly depicted. Nineteen (86,4%) out of 22 MCA aneurysms were visualized; an overview of 3D-US angiography images is shown in Fig. 2, and the images of the single posterior communication artery aneurysm are shown in Fig. 3.

We did not observe any association between the aneurysm visibility and its rupture (i.e. presence of SAH) or its size in our study group. In the subgroup of 22 MCA aneurysms, no association between aneurysm visibility and its rupture was observed; a trend towards insufficient sonographic visibility in smaller aneurysms was found (*p* = 0.09).

Based on the assessment of the 3D-US PD angiography screenshots and review of patients’ operative reports, the potential causes for the insufficient/absent 3D-US aneurysm visualization were identified. In three MCA aneurysms, the reasons were as follows: the PD gain set too high, resulting in displaying noise artifacts (case No. 13); the ultrasound signal of an MCA bifurcation aneurysm (case No. 18) merged with the signal of the adjacent M2 branch due to a very small aneurysm size (the aneurysm was depicted only hinted); in case No. 20, the new 3D-US system was used for the first time during aneurysm surgery, and the M4 aneurysm was not visualized due to improper power-Doppler gain and incorrect depth setting (after correct setting, the shallow sulcus containing the superficial M4 aneurysm was located using 2D-US and no 3D-US data acquisition was needed). In the case of a posterior inferior cerebellar artery aneurysm operated in a sitting position, the insufficient ultrasound scanning was due to air trapped in the basal subarachnoid cisterns (No. 5); in the case of an anterior communicating artery (AComA) aneurysm, the surgeon used PD angiography only after clipping in order to evaluate the flow in the A2 segments of both anterior cerebral arteries (No. 22).

### 3D-US PD angiography usage

The lack of a control group and the retrospective character of this study assessing ultrasonic aneurysm visibility did not allow additional quantification of the “added value” of 3D-US angiography; surgical findings concerning the 3D-US utilization were summarized as recorded in the official surgical reports:

#### Ruptured cases

**Fig. 4:**
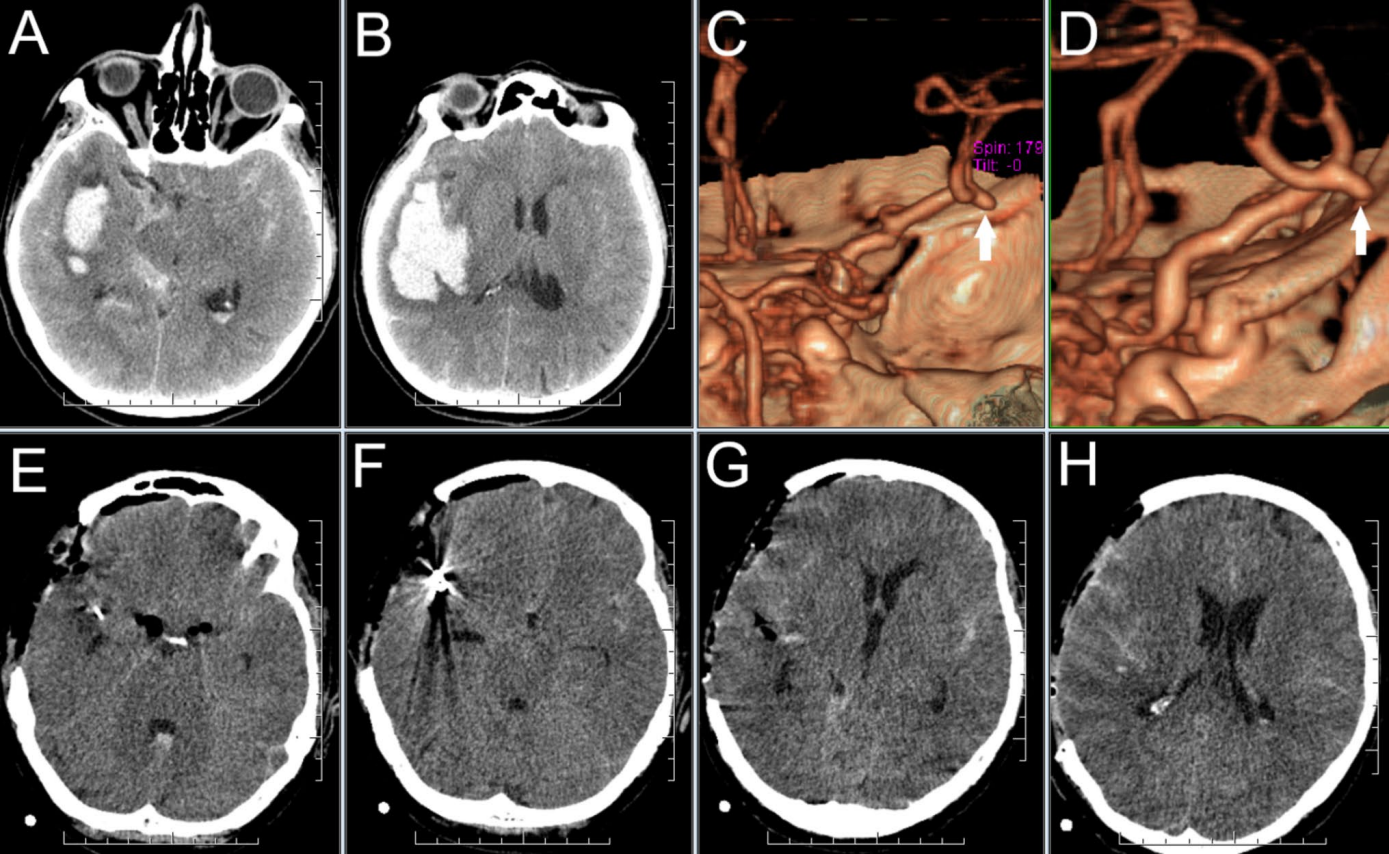
**Ruptured right-sided MCA aneurysm** – case No. 10 **A** and **B**: Preoperative CT showing massive subarachnoid hemorrhage and large right sided temporal hematoma extending into deeper brain structures. **C and D:** 3D reconstruction of CT angiography showing right-sided MCA aneurysm directed to the temporal lobe.** E – H** CT images performed on the first postoperative day

**Fig. 5:**
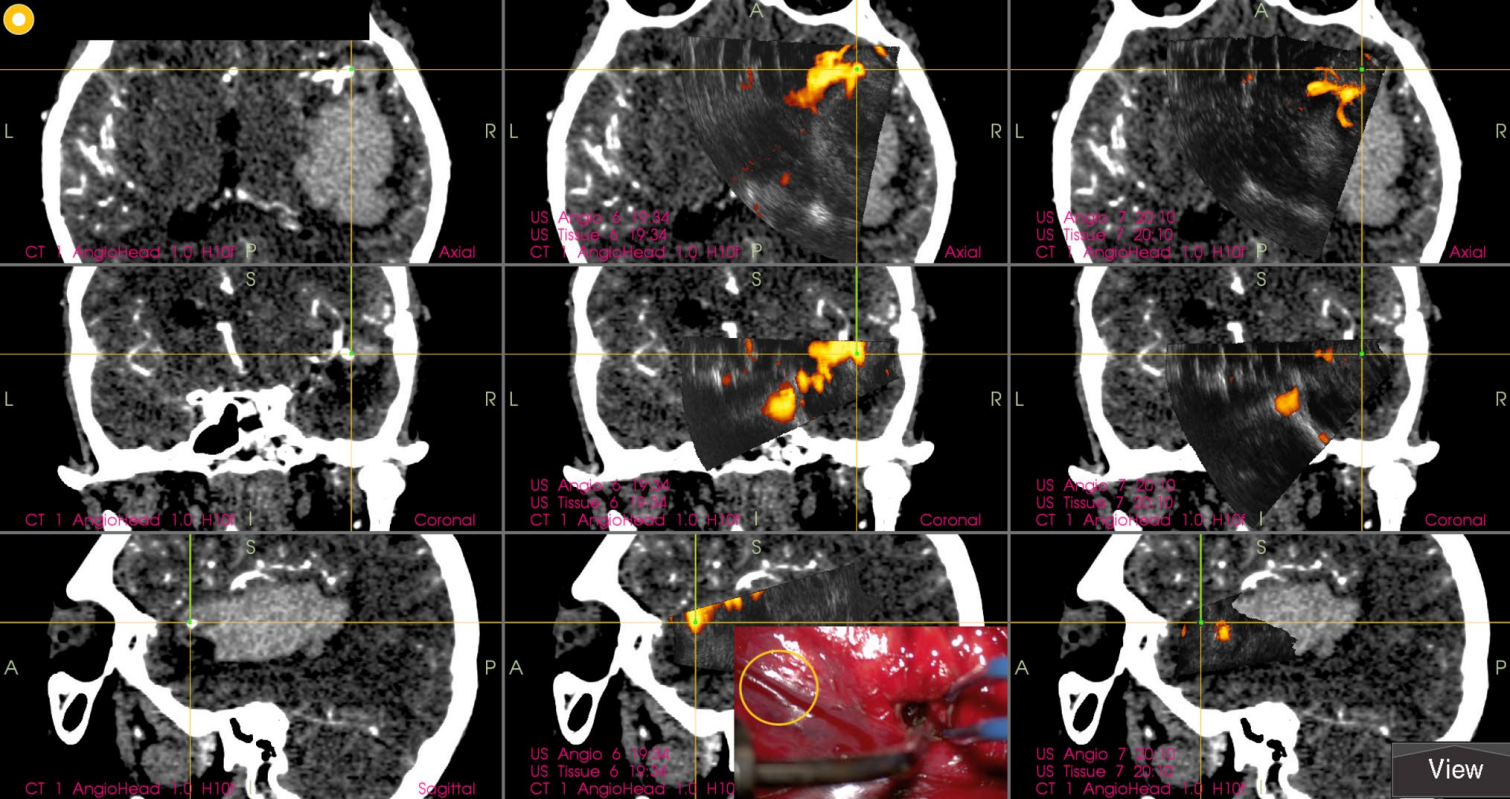
**3D-US images of case No. 10. Left column:**Preoperative navigation CT angiography. **Middle column:**Initial 3D-US power-Doppler image acquired before the start of hematoma evacuation, note the aneurysm is well visible in the same place as depicted by preoperative CT. *Inset:*After acquisition of initial 3D-US scans and localization of the (dangerous) area in the vicinity of the aneurysm, partial hematoma evacuation at a safe distance was performed C).  **Right column:** 3D-US power-Doppler image acquired after partial hematoma evacuation, note the significant shift of the aneurysm. The updated 3D-US image allowed identification of aneurysm position and helped to prevent hematoma evacuation too close to the aneurysm and rebleeding

**Fig. 6:**
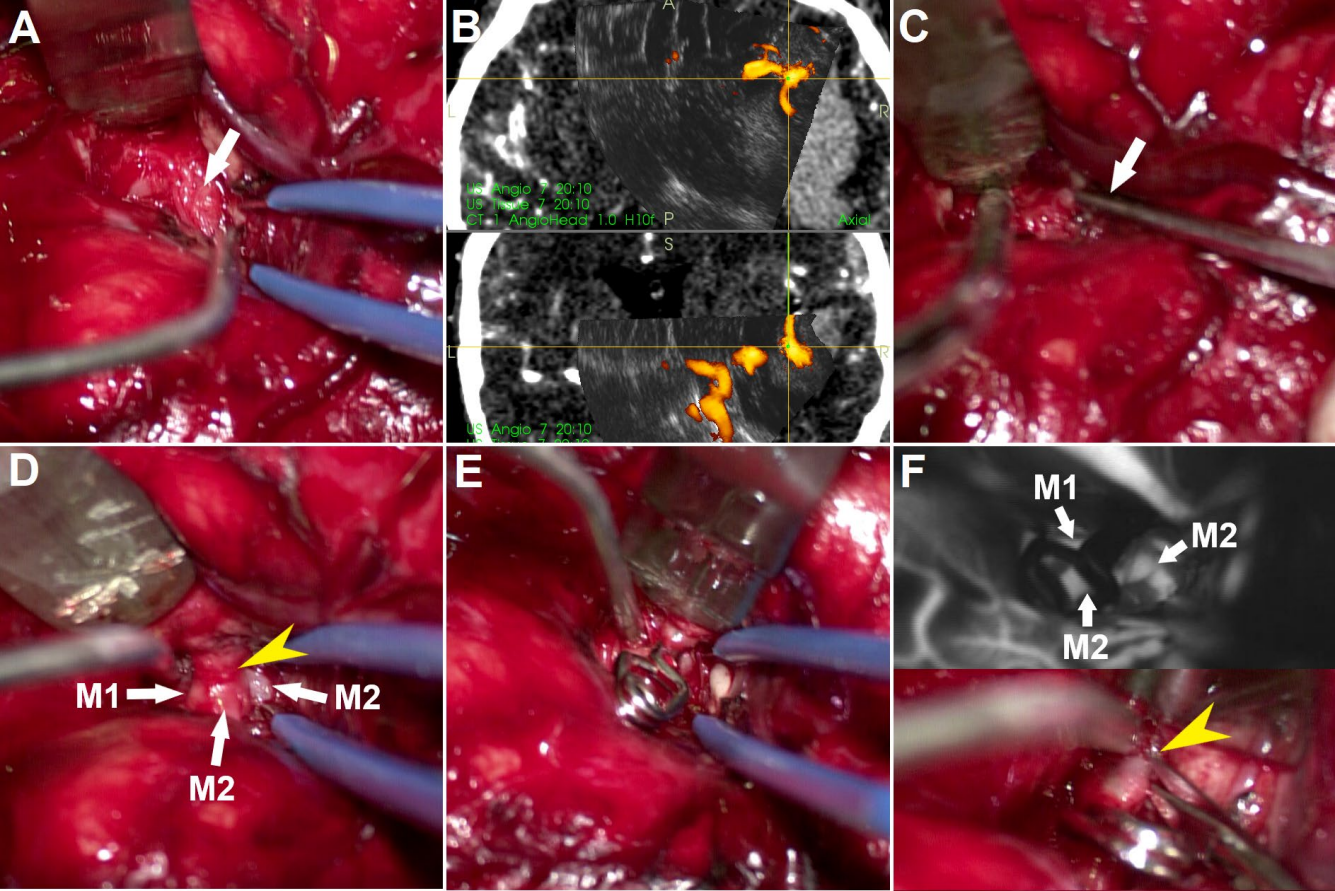
**Intraoperative photographs of case No. 10. A: **Early stages of Sylvian fissure dissection after partial hematoma evacuation. Note the scar tissue (arrow) surrounding the MCA branches.** B: **The updated 3D-US power-Doppler scan showing actual position of the aneurysm. Center of the yellow cross is located in the area of the aneurysm neck. **C: **Aneurysm neck localized within the scar tissue by pointer using the updated 3D-US images (arrow).** D: **Situation after deliberating MCA branches and aneurysm neck (yellow arrowhead) from the scar tissue. M1 = M1 segment of MCA, M2 = M2 segment of MCA. **E: **Visual inspection after aneurysm clipping.** F: **Test of aneurysm occlusion. *Upper image:*indocyanine green video angiography confirming complete aneurysm occlusion and blood flow in parent and branching vessels. M1 = M1 segment of MCA, M2 = M2 segment of MCA.  *Lower image:*exposing the aneurysm dome before final confirmation of the completeness of aneurysm occlusion by incising aneurysm dome. Yellow arrowhead = site of aneurysm rupture

**Fig. 7:**
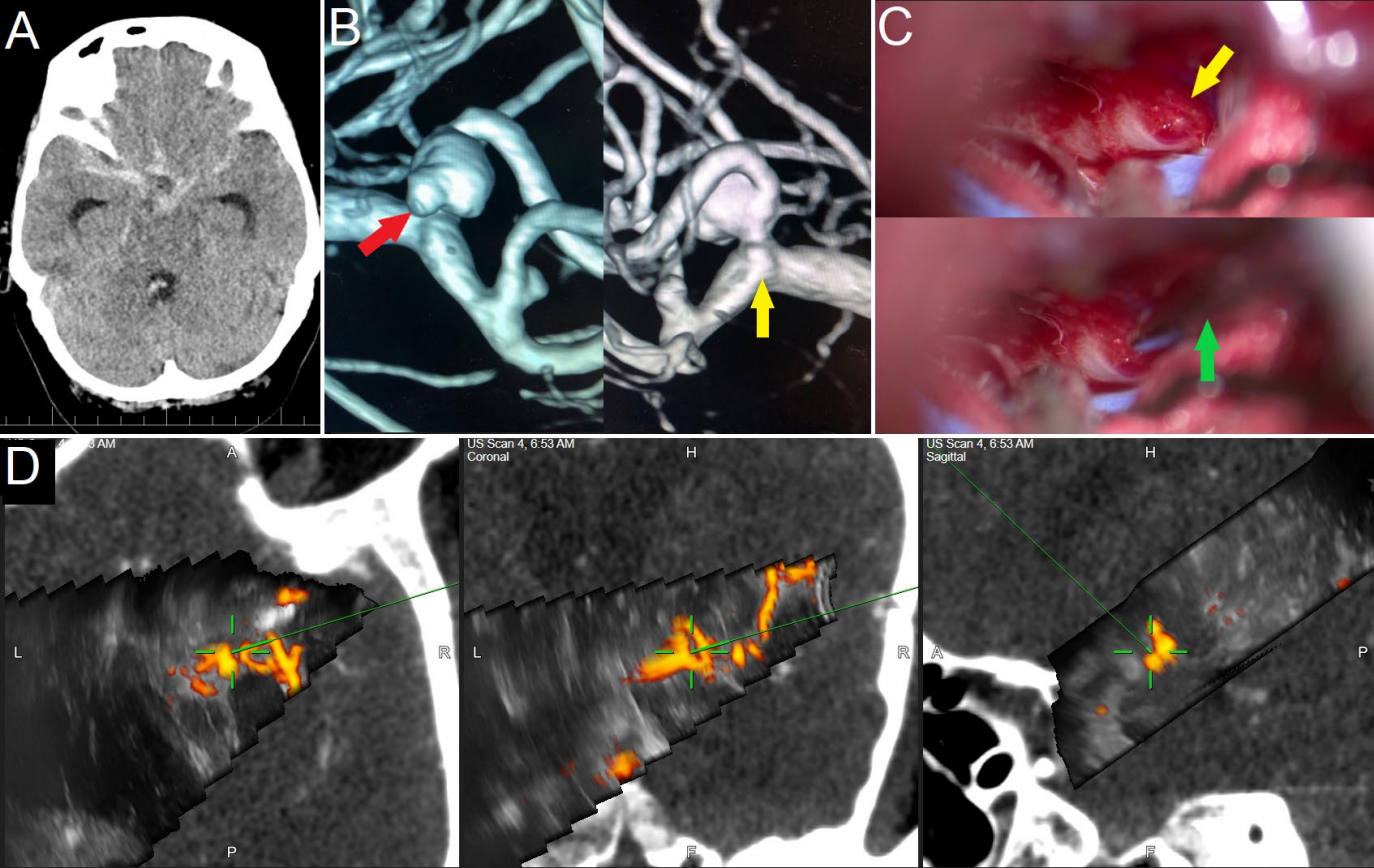
**Ruptured right-sided MCA aneurysm – case No. 25. A: **Preoperative CT showing massive subarachnoid hemorrhage (modified Fisher scale grade IV.). **B:** 3D reconstructions of rotational DSA, presumed site of the rupture was the aneurysm daughter sac *(red arrow)*. In addition, a bleb in the aneurysm neck was depicted as well *(yellow arrow)*. Due to the aneurysm morphology the endovascular treatment was refused by interventional radiologists.** C: **Intraoperative photographs. *Upper image: *A small extremely thin-walled blister aneurysm *(yellow arrow)* was found during Sylvian fissure dissection. *Lower image:* Navigation pointer (green arrow) was used for better anatomical orientation. **D:** Updated 3D-US power-Doppler scan confirming the location of the blister aneurysm is in the MCA bifurcation. Subsequent manipulation with MCA was considered to be unacceptably dangerous; the patient was subsequently treated endovascularly on a delayed basis after 2 weeks of controlled normotension

Preoperative radiological data and data on intraoperative 3D-US PD angiography utilization described in the official operative reports of 15 surgeries of ruptured aneurysms are summarized in Table 2. Presence of an intrasylvian hematoma (ISH) was defined based on the criteria by Fukuda et al. as a massive hematoma centering on the sylvian fissure and measuring longer than 15 millimeters (mm) along the short axis between the insula and the temporal lobe [12]. According to the reviewed operative reports, the most common uses of 3D-US PD angiography application in the subgroup of our patients with ruptured aneurysms were the identification of the exact position of aneurysms and surrounding vasculature (using a navigation pointer) in the presence of a significant brain-shift, large intracerebral hematomas (ICHs), ISHs, and extensive scars and adhesions; as well as the possibility of quick improvement of the surgeon’s mental 3D representation of the actual anatomical situation. The aforementioned 3D-US utilization helped us to perform surgical steps such as evacuation of ICHs and ISHs, distal Sylvian fissure dissection or dissection in the vicinity of the aneurysm more safely (Figs. 4, 5, 6, and 7 ).

**Table 2:** Preoperative radiological data and data on intraoperative 3D-US PD angiography utilization during 15 surgeries of ruptured aneurysms. Acom = Anterior communicating artery, cm = centimeters, CSF = cerebrospinal fluid, ICH = intracerebral hematoma, ISH = intrasylvian hematoma, IVH = Intraventricular Hemorrhage, MCA = Middle cerebral artery, mm = millimeters, Pcom = posterior communicating artery, SAH = subarachnoid hemorrhage, SDH = subdural hematoma

Case No.	Aneurysm location	Intracranial bleeding type	Intraoperative ultrasound use (as described in official surgical reports)
1.	MCA	SAH	Facilitating localization of the aneurysm sac in the blood-filled Sylvian fissure
2.	MCA	SAH	Facilitating localization of the aneurysm sac in the blood-filled Sylvian fissure
8.	MCA	SAH	No specific of 3D-US use mentioned
10.	MCA	SAH, ICH (volume = 57 cm^3^)	Verification of the position of the ICH, MCA branches and aneurysm; subsequent partial evacuation of the ICH; localization of the aneurysm after its shift
11.	MCA	SAH, ICH (volume = 28.5 cm^3^)	Verification of the position of the ICH, MCA branches and aneurysm; subsequent partial evacuation of the ICH; localization of the aneurysm after its shift; identification of the aneurysm neck within extensive scars (Figs. 4, 5 and 6)
12.	MCA	SAH, ICH (volume = 10 cm^3^)	Verification of the position of the ICH and aneurysm after significant CSF evacuation and brain-shift
14.	MCA	SAH, ISH (short axis length = 18 mm)	Verification of the actual aneurysm position before distal Sylvian fissure dissection and partial evacuation of the ISH
17.	MCA	SAH, ICH (volume = 52.5 cm^3^), IVH	Verification of the actual position of the aneurysm and subsequent partial ICH evacuation
18.	MCA	SAH, ISH (short axis length = 16 mm)	3D-US navigated ventricular puncture and CSF evacuation after obstruction of the lumbar drainage. Facilitating localization of the aneurysm neck within the blood-filled Sylvian fissure with extensive adhesions (aneurysm image was suboptimal, only aneurysm neck was visible)
20.	MCA	SAH, ICH (volume 16.5 = cm^3^)	Identification of the superficial M4 aneurysm within a shallow sulcus using 2D-ultrasound PD mode (3D-US aneurysm imaging was insufficient)
21.	Pcom	SAH, ICH (volume 5.2 = cm^3^), IVH, SDH	Verification of the aneurysm position, facilitating localization of the aneurysm neck within adhesions (Fig. 3)
22.	Acom	SAH, ICH (volume 28 = cm^3^), IVH	Bilateral PD verification of the blood-flow within anterior cerebral artery branches after the clipping. (No 3D-US aneurysm imaging was performed, ultrasound was used only after the aneurysm clipping)
23.	MCA	SAH	Verification of the actual aneurysm position and subsequent distal Sylvian fissure dissection
24.	MCA	SAH, ICH (volume 40 = cm^3^), IVH	Visualization of significant ICH volume enlargement - early rebleeding in the period between CT investigation and durotomy occurred. 3D-US evaluation of the extent of hematoma evacuation and verification of the blood-flow within MCA branches after the clipping
25.	MCA	SAH	Early identification of the location of a thin-walled blister aneurysm in the MCA bifurcation - likely preventing its rupture (Fig. 7)

#### Unruptured cases

**Fig. 8:**
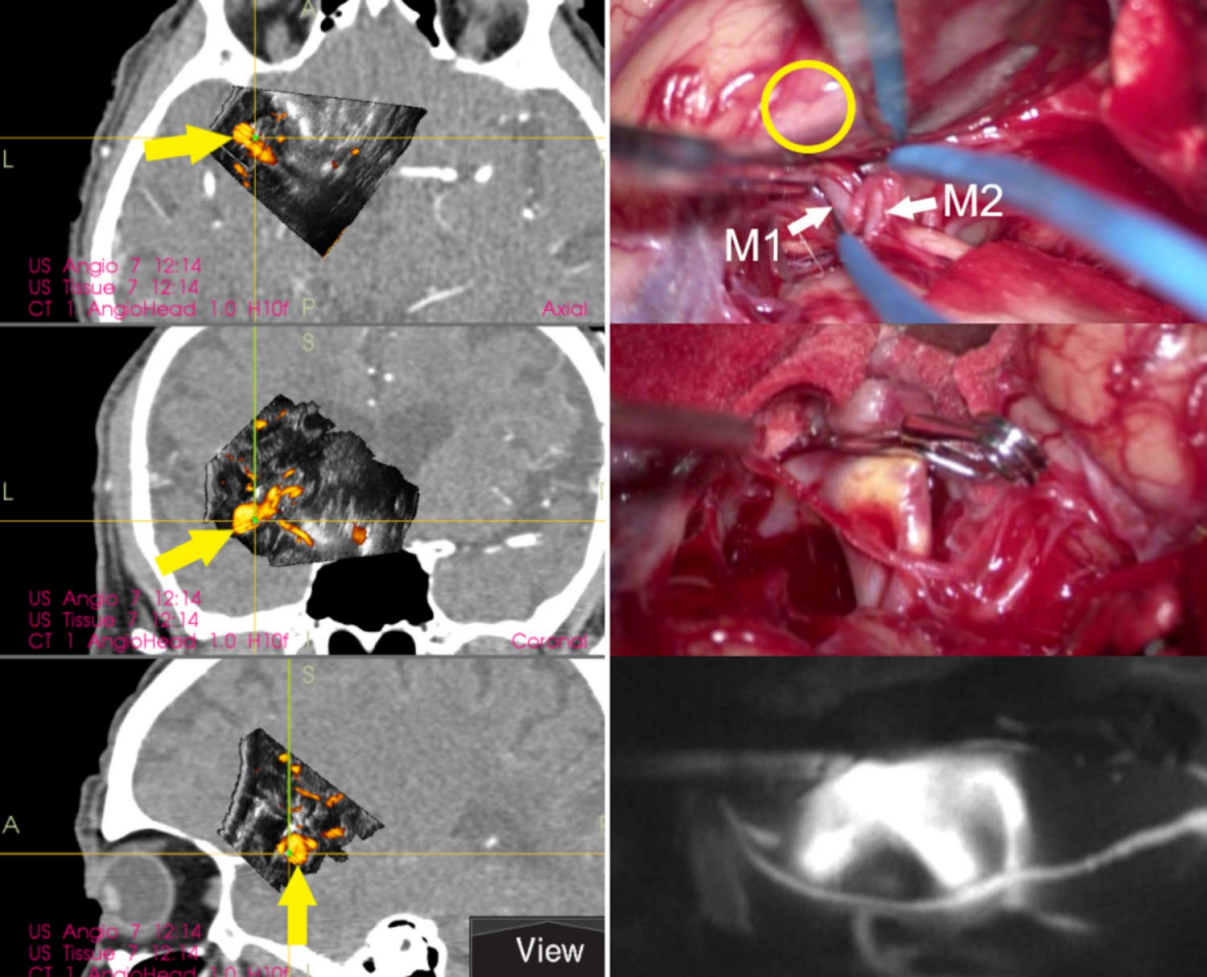
**Unruptured left-sided MCA aneurysm – case No. 14. Left column: **Navigated 3D-US scan performed after partial dissection of the Sylvian fissure, note the MCA aneurysm directing to the temporal lobe. **Right column:**Intraoperative photographs. *Upper image: *Situation shortly before the completion of Sylvian fissure dissection, no parts of aneurysm are visible. Yellow circle = presumed area of aneurysm dome as detected according to updated 3D-US images. Note the retractor is deliberately placed in position avoiding pressure on the brain tissue adjacent to the aneurysm. M1 = M1 segment of MCA, M2 = M2 segment of MCA.  *Middle image: *Situation after aneurysm clipping. *Lower image:*Indocyanine green video angiography confirming complete aneurysm occlusion and blood flow in M1 and M2 segments of MCA

The use of 3D-US PD angiography was described in 7 out of 10 operative reports. According to the reviewed operative reports, the ability of 3D-US to precisely localize aneurysms during dissection (prior to exposure of any aneurysm part) was described as useful, e.g., in the presence of adhesions/scars (case No 19) or significant brain-shift (case No 15, Fig. 8); faster dissection (as a result of a quick improvement of the 3D mental image) was also described (case No 7).

## Discussion

To the best of our knowledge, despite the relatively low number of cases in our study, so far it represents the largest reported series of cerebral aneurysms surgeries performed using navigated 3D-US PD angiography. Its utilization during aneurysm surgeries has so far been described only in two peripheral [36, 68] and one bifurcation MCA aneurysm [45], as well as in five aneurysms cases without a closer definition of their location [40]. In seven out of these eight reported cases the aneurysms were successfully visualized; of note, only three aneurysms were shown in published images [36, 45, 68]. This limited amount of data allowed only a very approximate idea of the possibilities of visualizing cerebral aneurysms by means of navigated 3D-US PD angiography. Interestingly, unlike in aneurysm surgeries, the benefits of navigated 3D-US PD angiography used during the surgical treatment of arteriovenous malformations (AVMs) have been analyzed in scientific studies [34, 67, 69].

Numerous reports describing the intraoperative use of 2D-US aneurysm imaging based on Doppler mode [4, 6, 26, 32, 35, 38, 70] or contrast-enhanced ultrasound [1, 10, 16, 37, 41] have been published. However, a disadvantage of all 2D-US systems is that the ultrasound image is present only during scanning, and it might be challenging to identify the exact position of smaller structures, e.g., the aneurysm neck, when an ultrasound probe is covering a substantial portion of the operating field [68]. This drawback is minimized by navigated 3D-US angiography, which enables identification of the actual position of the aneurysm and/or surrounding vessels by using a pointer [68].

In our series, we were able to visualize aneurysms in 20 out of 25 cases (80%). While we did not observe any association between the sonographic aneurysm visibility and aneurysm size or the presence of SAH, our case-by-case assessment of the causes of the insufficient imaging of the five aneurysms with suboptimal/absent visualization indicates that in four aneurysms the cause was most probably an erroneous ultrasound setting or improper 3D-US utilization (and not an insufficiency of the hardware or software parameters of the 3D-US systems). In the fifth case, however, PD blooming caused merging of the PD signal of the small MCA bifurcation aneurysm with the PD signal of the adjacent M2 branch; indeed, in our subgroup of 22 MCA aneurysms, a trend towards insufficient sonographic visibility in smaller aneurysms was found.

Importantly, as 22 out of 25 of the cases (88%) were MCA aneurysms, it is not possible to draw conclusions regarding the 3D sonographic visibility of aneurysms in other locations. Nevertheless, as two out of three non-MCA aneurysms were not depicted using 3D-US angiography, we decided to include all aneurysms (and not to present only the MCA aneurysms subgroup); more presented 3D-US scanning pitfalls may be of higher information value.

### Subgroup of MCA aneurysms

In our series, PD mode was sensitive enough to visualize the vast majority of MCA aneurysms (86,4%). Nevertheless, to achieve 3D-US PD angiography images of sufficient quality, care must be taken during the ultrasound scanning; the freehand probe movements should be performed very slowly, as gentle movement of the probe may significantly reduce the flash artifacts [47].

In our series, there were two reasons why MCA aneurysms were much more common than aneurysms in other locations. First, in our series of operated aneurysms, the MCA aneurysms were more common than the aneurysms at all other locations. Second, as the majority of MCA aneurysms are located relatively close to the brain surface [30], they are prone to a considerable shift [15]; using a sophisticated algorithm, Reinertsen et al. examined the positions of different parts of a bifurcation MCA aneurysm before dissection and found an average shift of the aneurysm by more than seven mm compared to its position on preoperative MRI angiography [45]. Brain swelling, large hematomas, or substantial evacuation of cerebrospinal fluid may contribute to more extensive and clinically relevant shift of MCA aneurysms [50, 53]. Interestingly, in the study conducted by Rohde et al. the main purpose of applying navigation in the MCA aneurysm subgroup, comprising M1 segment aneurysms and MCA bifurcation aneurysms, was to localize the lesion (in the AComA aneurysm subgroup, the main purpose of applying navigation was to better understand the branching anatomy during the surgery) [46].

Based on a retrospective assessment of our subgroup of MCA aneurysms, identifying the exact position of the MCA aneurysms seemed to be useful during the evacuation of ICHs and ISHs, helping to avoid the removal of too much blood clot and to prevent intraoperative bleeding from the aneurysm. Similarly, 3D-US PD angiography seemed to be helpful in localizing the position of the aneurysm dome and neck when the dissection was complicated by blood-clots within the Sylvian fissure and/or by scars after previous (undiagnosed) SAH. Additionally, we found the possibility to compensate for the brain-shift and to improve surgeon’s mental 3D representation of the aneurysm and surrounding vessels based on updated 3D-US angiography to be helpful (similar considerations have already been labelled in reports on aneurysm [39, 46] and AVM surgeries [34]). Nevertheless, potential clinical relevance of our initial observations has to be confirmed in prospective studies.

### Study limitations

Undoubtedly, our results have to be taken with caution. First of all, the utilization of navigation during aneurysm surgeries – both conventional as well as updatable by intraoperative imaging modalities – is a relatively controversial topic, and many cerebrovascular centers do not use navigation during aneurysm surgery. Nevertheless, its utilization during surgeries of distal aneurysm has been reported repeatedly [11, 14, 24, 29, 35, 50] and is relatively well-known. However, numerous centers described the use of navigation (conventional or updatable) during surgeries of non-distal aneurysms as well [7, 8, 23, 31, 39, 43, 46, 53, 57, 65, 66]. Some centers use navigation during aneurysm surgeries routinely, as it may improve safety – although its benefit may often be limited, sometimes it can be of significant help, e.g., in targeting the Sylvian fissure opening, identifying where vessels branch from the aneurysm base, locating the site of rupture, daughter blebs, or even adherent vessels, etc. (personal communication with Prof. A. Raabe). The second drawback of our study is the fact that the majority of the cases (88%) were MCA aneurysms. Nevertheless, we believe that information concerning MCA aneurysm visualization (which was evaluated also separately) may be of interest – large ICHs distorting anatomy, which require immediate evacuation [3], are more common after the rupture of MCA aneurysms than after the rupture of aneurysms in other location [9]. In addition, clear knowledge of the exact localization and projection of the MCA aneurysm dome might facilitate precise dissection in a blood-filled Sylvian fissure [46]; extensive evacuation of firm ISH clots may be demanding and risky [64]. Thirdly, we did not compare 3D aneurysm-anatomy details (e.g., small daughter sacs) visualized by 3D-US with other imaging modalities (a special program would be needed). Fourthly, the retrospective character of the study represents a significant study limitation on its own. Lastly, the overall number of 83 aneurysm patients operated in our center during a period of seven years is low (in Slovakia the vast majority of aneurysms are referred for endovascular treatment by neurologists, without an obligation to consult a neurosurgeon). It is likely that in centers with a higher number of clipped aneurysms, 3D-US PD angiography may be less helpful, as the surgical expertise tends to grow with the number of operated cases.

### Implications for potential future research

Due to the decreasing number of aneurysms treated surgically, training and maintenance of neurosurgical skills is becoming more and more challenging [21], especially in regions with a high prevalence of endovascular treatment [54, 55]. The number of neurosurgeons capable of clipping even simple aneurysms is decreasing [49]. Young neurosurgeons report difficulties in creating a 3D mental image during dissection around unruptured MCA aneurysms in situations where orientation for experienced vascular neurosurgeons would likely not be problematic [25]. Several centers, including our own, are nowadays facing the paradoxical situation where the same surgeons are required to perform often difficult dissection and clipping of ruptured aneurysms (many times with large hematomas) while not having enough opportunities to operate on simple and/or unruptured cases. Undoubtedly, the lesser experience with aneurysm surgeries makes the creation of the mental 3D representation of the (distorted) anatomical situation during dissection around the aneurysm more challenging; hence, some surgeons who are not specialized cerebrovascular experts might benefit from having a tool allowing visualization of the actual vascular anatomy during the entire procedure. Especially surgical steps described in the literature as potentially challenging, e.g., a hematoma evacuation (which can cause premature aneurysm rupture) [28], identification of an aneurysm and vascular structures in situations when these are covered by blood clots or edematous brain tissue [9, 43, 46], distinguishing an M2 trunk with medial course from the M1 segment [30], or distinguishing whether the first visible M2 segment is the upper or lower trunk in the presence of massive SAH [17], could understandably present potential pitfall for less experienced surgeons. However, well-designed prospective studies are needed to reveal if navigated 3D-US PD angiography could be of additional value in similar situations and whether it has the potential to reduce surgical time or complication rates.

The benefits of updatable navigation have also been emphasized by some prominent cerebrovascular centers. As pointed out by Raabe and colleagues, in some situations (e.g., the presence of massive blood clots or brain edema), even with the best anatomical knowledge, a surgeon may not always be sure about the location of the vessels with respect to the aneurysm [43]. Hence, it can be implicitly assumed that in some centers, where no intraoperative imaging enabling navigation data update is being used, certain benefits of navigated 3D-US angiography might be identified. However, again, only future studies may confirm or refute this hypothesis.

Lastly, while we did not systematically use the updated 3D-US B-mode images to illustrate the final degree of ICH evacuation (excessive pursuit of ICH evacuation can lead to neurovascular damage [71]), the newest data show that in some cases intraoperative CT may be necessary to avoid unexpectedly large hematoma residuals [3]. Again, only prospective studies may verify if navigated 3D-US imaging may be successfully used for increasing the degree of safe hematoma evacuation.

## Conclusions

In our subgroup of MCA aneurysms, the navigated 3D-US PD angiography imaging was satisfactory in vast majority of cases. The small size of aneurysms appears to be a factor that negatively affects their sonographic visibility. Future prospective studies with a sufficient number of aneurysms localized at various anatomic sites are needed to confirm our primary results. In addition, further research is needed to establish (or refute) the potential added value of this intraoperative imaging method.

## Data Availability

No datasets were generated or analysed during the current study.

## Abbreviations

2D: US 2D-ultrasound
3D: US 3D-ultrasound
AComA: Anterior communicating artery
AVM: Arteriovenous malformations
B-mode: Brightness mode
CT: Computed tomography
DSA: Digital subtraction angiography
MCA: Middle cerebral artery
mm: millimeters
MRI: Magnetic resonance imaging
PD: power-Doppler
SAH: Subarachnoid hemorrhage

## References

[1] Acerbi F, Prada F, Vetrano IG, Falco J, Faragò G, Ferroli P, DiMeco F (2019) Indocyanine green and contrast-enhanced ultrasound videoangiography: a synergistic approach for real-time verification of distal revascularization and aneurysm occlusion in a complex distal middle cerebral artery aneurysm. World Neurosurg 125:277–284. 10.1016/j.wneu.2019.01.241 30776513 10.1016/j.wneu.2019.01.241

[2] Aleo D, Elshaer Z, Pfnür A, Schuler PJ, Fontanella MM, Wirtz CR, Pala A, Coburger J (2022) Evaluation of a navigated 3D ultrasound integration for brain tumor surgery: first results of an ongoing prospective study. Curr Oncol (Toronto Ont) 29(9):6594–6609. 10.3390/curroncol29090518 10.3390/curroncol29090518 PMC9498154 36135087

[3] Autio AH, Paavola J, Tervonen J, Lång M, Elomaa AP, Huuskonen TJ, Huttunen J, Kärkkäinen V, Zu FraunbergLindgren MAE, Koivisto T, Kurola J, Jääskeläinen JE, Kämäräinen OP (2024) Acute evacuation of 54 intracerebral hematomas (aICH) during the microsurgical clipping of a ruptured middle cerebral artery bifurcation aneurysm-illustration of the individual clinical courses and outcomes with a serial brain CT/MRI panel until 12 months. Acta Neurochir 166(1):17. 10.1007/s00701-024-05902-9 38231317 10.1007/s00701-024-05902-9 PMC10794262

[4] Barr LL, Babcock DS, Crone KR, Berger TS, Ball WS, Prenger EC (1991) Color Doppler US imaging during pediatric neurosurgical and neuroradiologic procedures. Radiology 181:567–571. 10.1148/radiology.181.2.1924806 1924806 10.1148/radiology.181.2.1924806

[5] Bekedam NM, Karssemakers LHE, van Alphen MJA, van Veen RLP, Smeele LE, Karakullukcu MB (2023) Comparison of image quality of 3D ultrasound: motorized acquisition versus freehand navigated acquisition, a phantom study. Int J Comput Assist Radiol Surg 18(9):1649–1663. 10.1007/s11548-023-02934-x 37243918 10.1007/s11548-023-02934-x PMC10491552

[6] Black KL, Rubin JM, Chandler WF, McGillicuddy JE (1988) Intraoperative color-flow doppler imaging of AVM’s and aneurysms. J Neurosurg 68:635–639. 10.3171/jns.1988.68.4.0635 3280749 10.3171/jns.1988.68.4.0635

[7] Carl B, Bopp M, Chehab S, Bien S, Nimsky C (2018) Preoperative 3-dimensional angiography data and intraoperative real-time vascular data integrated in microscope-based navigation by automatic patient registration applying intraoperative computed tomography. World Neurosurg 113:e414–e425. 10.1016/j.wneu.2018.02.045 29454128 10.1016/j.wneu.2018.02.045

[8] Chibbaro S, Tacconi L (2006) Image-guided microneurosurgical management of vascular lesions using navigated computed tomography angiography. An advanced IGS technology application. Int J Med Robot 2:161–167. 10.1002/rcs.89 17520627 10.1002/rcs.89

[9] Darkwah Oppong M, Skowronek V, Pierscianek D, Gembruch O, Herten A, Saban DV, Dammann P, Forsting M, Sure U, Jabbarli R (2018) Aneurysmal intracerebral hematoma: risk factors and surgical treatment decisions. Clin Neurol Neurosurg 173:1–7. 10.1016/j.clineuro.2018.07.014 30053744 10.1016/j.clineuro.2018.07.014

[10] Del Bene M, DiMeco F, Prada F (2016) Contrast-enhanced Ultrasound (CEUS) in neurosurgery. In: Prada F, Solbiati L, Martegani A, DiMeco F (eds) Intraoperative ultrasound (IOUS) in neurosurgery—from standard B-mode to elastosonography. Springer, Cham Heidelberg New York Dordrecht London, pp 135–145

[11] Fierstra J, Anon J, Mendelowitsch I, Fandino J, Diepers M, Remonda L, Marbacher S (2020) Amended intraoperative neuronavigation: three-dimensional vascular roadmapping with selective rotational digital subtraction angiography. World Neurosurg 135:183–187. 10.1016/j.wneu.2019.12.055 31863893 10.1016/j.wneu.2019.12.055

[12] Fukuda H, Hayashi K, Moriya T, Nakashita S, Lo BW, Yamagata S (2015) Intrasylvian hematoma caused by ruptured middle cerebral artery aneurysms predicts recovery from poor-grade subarachnoid hemorrhage. J Neurosurg 123(3):686–692. 10.3171/2014.10.JNS141658 26052880 10.3171/2014.10.JNS141658

[13] Gläsker S, Shah MJ, Hippchen B, Neumann HP, van Velthoven V (2011) Doppler-sonographically guided resection of central nervous system hemangioblastomas. Neurosurgery 68(2 Suppl Operative):267–275. 10.1227/NEU.0b013e3182124677 21346656 10.1227/NEU.0b013e3182124677

[14] Hermann EJ, Petrakakis I, Götz F, Lütjens G, Lang J, Nakamura M, Krauss JK (2015) Surgical treatment of distal anterior cerebral artery aneurysms aided by electromagnetic navigation CT angiography. Neurosurg Rev 38:523–530. 10.1007/s10143-015-0611-9 25666391 10.1007/s10143-015-0611-9

[15] Hill DL, Maurer CR Jr, Maciunas RJ, Barwise JA, Fitzpatrick JM, Wang MY (1998) Measurement of intraoperative brain surface deformation under a craniotomy. Neurosurgery 43:514–526. 10.1097/00006123-199809000-00066 9733307 10.1097/00006123-199809000-00066

[16] Hölscher T, Ozgur B, Singel S, Wilkening WG, Mattrey RF, Sang H (2007) Intraoperative ultrasound using phase inversion harmonic imaging: first experiences. Neurosurgery 60(4 Suppl 2):382–387. 10.1227/01.NEU.0000255379.87840.6E 17415178 10.1227/01.NEU.0000255379.87840.6E

[17] Ikawa F (2019) Surgery of Middle cerebral artery (MCA) Aneurysm. In: July J, Wahjoepramono EJ (eds) *Neurovassurgeryurgery*: Surapproachesoaches for Neurovasdiseasesseases. Springer, Singapore, pp 125–134

[18] Jakola AS, Myrmel KS, Kloster R, Torp SH, Lindal S, Unsgård G, Solheim O (2012) Comparison of a strategy favoring early surgical resection vs a strategy favoring watchful waiting in low-grade gliomas. JAMA 308:1881–1888. 10.1001/jama.2012.12807 23099483 10.1001/jama.2012.12807

[19] Jakola AS, Skjulsvik AJ, Myrmel KS, Sjåvik K, Unsgård G, Torp SH, Aaberg K, Berg T, Dai HY, Johnsen K, Kloster R, Solheim O (2017) Surgical resection versus watchful waiting in low-grade gliomas. Ann Oncol 28:1942–1948. 10.1093/annonc/mdx230 28475680 10.1093/annonc/mdx230 PMC5834105

[20] Jayakumar PN, Ravishankar S, Balasubramaya KS, Chavan R, Goyal G (2007) Disappearing saccular intracranial aneurysms: do they really disappear? Interv Neuroradiol 13(3):247–254. 10.1177/159101990701300304 20566116 10.1177/159101990701300304 PMC3345488

[21] Joseph FJ, Vanluchene HER, Bervini D (2023) Simulation training approaches in intracranial aneurysm surgery-a systematic review. Neurosurg Rev 46:101. 10.1007/s10143-023-01995-5 37131015 10.1007/s10143-023-01995-5 PMC10154262

[22] Kato Y, Nouri M, Shu G (2019) Surgery of Anterior communicating artery aneurysms. In: July J, Wahjoepramono EJ (eds) *Neurovassurgeryurgery*: Surapproachesoaches for Neurovasdiseasesseases. Springer, Singapore, pp 117–124

[23] Khoshnevisan A, Abdollahzadeh S (2014) Neuronavigation for intraoperative localization of cerebral aneurysms. Arch Neurosci 1:e16164. 10.5812/archneurosci.16164

[24] Kim TS, Joo SP, Lee JK, Jung S, Kim JH, Kim SH, Kang SS, Yoon W (2007) Neuronavigation-assisted surgery for distal anterior cerebral artery aneurysm. Minim Invasive Neurosurg 50:140–144. 10.1055/s-2007-985151 17882748 10.1055/s-2007-985151

[25] Kimura Y, Mashiko T, Watanabe E, Kawai K (2021) Preoperative simulation of a middle cerebral artery aneurysm clipping using a rotational three-dimensional digital subtraction angiography. Surg Neurol Int 12:70. 10.25259/SNI_934_2020 33767874 10.25259/SNI_934_2020 PMC7982121

[26] Kondoff S, Alioski N, Spiriev T, Vassilev J, Simeonov G, Kostadinova C (2015) Intraoperative duplex sonography for the treatment of large and giant aneurysms. Retrospective analysis of 13 cases. Int J Surg Med 1:12–17. 10.5455/ijsm.189572

[27] Lashkarivand A, Ringstad G, Wiedmann M (2024) Empowering early career vascular neurosurgeons in the endovascular era: managing intraoperative aneurysm rupture through a systematic algorithmic approach. J Clin Neurosci 120:229–231. 10.1016/j.jocn.2024.01.006 38306902 10.1016/j.jocn.2024.01.006

[28] Lawton MT (2011) Seven aneurysms: tenets and techniques for clipping. Thieme, New York 10.1227/NEU.0b013e31821819b9 21389888

[29] Lee SH, Bang JS (2007) Distal middle cerebral artery M4 aneurysm surgery using Navigation-CT Angiography. J Korean Neurosurg Soc 42(6):478–480. 10.3340/jkns.2007.42.6.478 19096593 10.3340/jkns.2007.42.6.478 PMC2588183

[30] Lehecka M, Dashti R, Rinne J, Romani R, Kivisaari R, Niemelä M, Hernesniemi J (2012) Surgical Management of aneurysms of the Middle cerebral artery. In: Quinones-Hinojosa A (ed) Schmidek and Sweet Operative Neurosurgical techniques: indications, methods and results, vol 6. Elsevier, Philadelphia, pp 897–913

[31] Leng LZ, Rubin DG, Patsalides A, Riina HA (2013) Fusion of intraoperative three-dimensional rotational angiography and flat-panel detector computed tomography for cerebrovascular neuronavigation. World Neurosurg 79:504–509. 10.1016/j.wneu.2011.09.008 22120274 10.1016/j.wneu.2011.09.008

[32] Lindseth F, Lovstakken L, Rygh OM, Tangen GA, Torp H, Unsgaard G (2009) Blood flow imaging: an angle-independent ultrasound modality for intraoperative assessment of flow dynamics in neurovascular surgery. Neurosurgery 65(6 Suppl):149–157. 10.1227/01.NEU.0000345945.92559.C5 19934989 10.1227/01.NEU.0000345945.92559.C5

[33] Mason AM, Cawley CM, Barrow DL (2011) Surgical Management of Middle cerebral artery aneurysms. In: Winn HR (ed) Youmans neurological surgery, vol 6. Elsevier, Philadelphia, pp 3862–3870

[34] Mathiesen T, Peredo I, Edner G, Kihlström L, Svensson M, Ulfarsson E, Andersson T (2007) Neuronavigation for arteriovenous malformation surgery by intraoperative three-dimensional ultrasound angiography. Neurosurgery 60(4 Suppl 2):345–350. 10.1227/01.NEU.0000255373.57346.EC. (PMID: 17415173) 17415173 10.1227/01.NEU.0000255373.57346.EC

[35] Muhammad S, Zhang R, Filler T, Hänggi D, Meling TR (2024) Trans-lateral ventricular approach for surgical treatment of high-located P2-P3 junction posterior cerebral artery aneurysms: from anatomical research to clinical application. Acta Neurochir 166(1):50. 10.1007/s00701-024-05942-1 38289511 10.1007/s00701-024-05942-1 PMC10828004

[36] Nerntengian N, Gkasdaris G, Barettas N, Theodoropoulou E, Birbilis T (2021) The use of real-time 3d intraoperative ultrasound angiography in localization and occlusion control of a ruptured mycotic aneurysm: a case report. J Neurol Surg Cent Eur Neurosurg 82:500–504. 10.1055/s-0040-1720988 10.1055/s-0040-1720988 33278825

[37] Otsuki H, Nakatani S, Yamasaki M, Kinoshita A, Iwamoto F, Kagawa N (2001) Intraoperative ultrasound arteriography with the coded Harmonic Angio technique. Report of three cases. J Neurosurg 94:992–995. 10.3171/jns.2001.94.6.0992 11409531 10.3171/jns.2001.94.6.0992

[38] Payer M, Kaku Y, Bernays R, Yonekawa Y (1998) Intraoperative color-coded duplex sonography for localization of a distal middle cerebral artery aneurysm: technical case report. Neurosurg 1998 42:941–943. 10.1097/00006123-199804000-00153 10.1097/00006123-199804000-00153 9574663

[39] Pesce A, Frati A, D’Andrea G, Palmieri M, Familiari P, Cimatti M, Valente D, Raco A (2018) The real impact of an intraoperative magnetic resonance imaging-equipped operative theatre in neurovascular surgery: the Sapienza University experience. World Neurosurg 120:190–199. 10.1016/j.wneu.2018.08.124 30165208 10.1016/j.wneu.2018.08.124

[40] Policicchio D, Doda A, Sgaramella E, Ticca S, Veneziani Santonio F, Boccaletti R (2018) Ultrasound-guided brain surgery: echographic visibility of different pathologies and surgical applications in neurosurgical routine. Acta Neurochir 160:1175–1185. 10.1007/s00701-018-3532-x 29675718 10.1007/s00701-018-3532-x

[41] Prada F, Del Bene M, Saini M, Ferroli P, DiMeco F (2015) Intraoperative cerebral angiosonography with ultrasound contrast agents: how I do it. Acta Neurochir 157:1025–1029. 10.1007/s00701-015-2412-x 25854600 10.1007/s00701-015-2412-x

[42] Prada F, Del Bene M, Casali C, Saladino A, Legnani FG, Perin A, Moiraghi A, Richetta C, Rampini A, Mattei L, Vetrano IG, Fornaro R, Saini M, Martegani A, DiMeco F (2015) Intraoperative navigated angiosonography for Skull Base Tumor surgery. World Neurosurg 84(6):1699–1707. 10.1016/j.wneu.2015.07.025 26193670 10.1016/j.wneu.2015.07.025

[43] Raabe A, Beck J, Rohde S, Berkefeld J, Seifert V (2006) Three-dimensional rotational angiography guidance for aneurysm surgery. J Neurosurg 105:406–411. 10.3171/jns.2006.105.3.406 16961135 10.3171/jns.2006.105.3.406

[44] Raabe A, Fichtner J, Gralla J (2017) Advanced intraoperative imaging: gold standard in brain and spine surgery? Clin Transl Neurosci 1(1). 10.1177/2514183X17718312

[45] Reinertsen I, Lindseth F, Askeland C, Iversen DH, Unsgård G (2014) Intra-operative correction of brain-shift. Acta Neurochir 156:1301–1310. 10.1007/s00701-014-2052-6 24696180 10.1007/s00701-014-2052-6

[46] Rohde V, Hans FJ, Mayfrank L, Dammert S, Gilsbach JM, Coenen VA (2007) How useful is the 3-dimensional, surgeon’s perspective-adjusted visualisation of the vessel anatomy during aneurysm surgery? A prospective clinical trial. Neurosurg Rev 30:209–217. 10.1007/s10143-007-0076-6 17483972 10.1007/s10143-007-0076-6

[47] Rygh OM, Nagelhus Hernes TA, Lindseth F, Selbekk T, Brostrup Müller T, Unsgaard G (2006) Intraoperative navigated 3-dimensional ultrasound angiography in tumor surgery. Surg Neurol 66(6):581–592. 10.1016/j.surneu.2006.05.060 17145316 10.1016/j.surneu.2006.05.060

[48] Saftoiu A, Iordache S, Ciurea T, Dumitrescu D, Popescu M, Stoica Z (2005) Pancreatic pseudoaneurysm of the superior mesenteric artery complicated with obstructive jaundice. A case report. JOP 6(1):29–35 15650282

[49] Salaud C, Hamel O, Riem T, Desal H, Buffenoir K (2016) Management of aneurysmal subarachnoid haemorrhage with intracerebral hematoma: is there an indication for coiling first? Study of 44 cases. Interv Neuroradiol: J Peritherapeutic Neuroradiol Surg Procedures Relat Neurosci 22(1):5–11. 10.1177/1591019915617320 10.1177/1591019915617320 PMC4757382 26634802

[50] Sasao R, Takahashi S, Nishimoto M, Yoshida K (2018) Usefulness of Intraoperative Imaging in a patient with a ruptured aneurysm of the M4 segment of the Middle cerebral artery. World Neurosurg 120:90–95. 10.1016/j.wneu.2018.08.025 30121410 10.1016/j.wneu.2018.08.025

[51] Sastry R, Bi WL, Pieper S, Frisken S, Kapur T, Wells W 3rd Golby AJb (2017) applications of Ultrasound in the resection of brain tumors. J Neuroimaging: Official J Am Soc Neuroimaging 27(1):5–15. 10.1111/jon.12382 10.1111/jon.12382 PMC5226862 27541694

[52] Schaller K, Kotowski M, Pereira V, Rüfenacht D, Bijlenga P (2011) From intraoperative angiography to advanced intraoperative imaging: the geneva experience. Acta Neurochir Suppl 109:111–115. 10.1007/978-3-211-99651-5_18 20960330 10.1007/978-3-211-99651-5_18

[53] Schmid-Elsaesser R, Muacevic A, Holtmannspötter M, Uhl E, Steiger HJ (2003) Neuronavigation based on CT angiography for surgery of intracranial aneurysms: primary experience with unruptured aneurysms. Minim Invasive Neurosurg 46:269–277. 10.1055/s-2003-44455 14628242 10.1055/s-2003-44455

[54] Skodvin TØ, Kloster R, Sorteberg W, Isaksen JG (2021) Survey of European neurosurgeons’ management of unruptured intracranial aneurysms: inconsistent practice and organization. Acta Neurochir 163:113–121. 10.1007/s00701-020-04539-8 32870423 10.1007/s00701-020-04539-8 PMC7778617

[55] Smith GA, Dagostino P, Maltenfort MG, Dumont AS, Ratliff JK (2011) Geographic variation and regional trends in adoption of endovascular techniques for cerebral aneurysms. J Neurosurg 114:1768–1777. 10.3171/2011.1.JNS101528 21314274 10.3171/2011.1.JNS101528

[56] Solberg OV, Lindseth F, Torp H, Blake RE, Nagelhus Hernes TA (2007) Freehand 3D ultrasound reconstruction algorithms–a review. Ultrasound Med Biol 33(7):991–1009. 10.1016/j.ultrasmedbio.2007.02.015 17512655 10.1016/j.ultrasmedbio.2007.02.015

[57] Son YJ, Han DH, Kim JE (2007) Image-guided surgery for treatment of unruptured middle cerebral artery aneurysms. Neurosurgery 61(5 Suppl 2):266–272. 10.1227/01.neu.0000303979.88880.06 18091241 10.1227/01.neu.0000303979.88880.06

[58] Šteňo A, Buvala J, Babková V, Kiss A, Toma D, Lysak A (2021) Current limitations of Intraoperative Ultrasound in Brain Tumor surgery. Front Oncol 11:659048. 10.3389/fonc.2021.659048 33828994 10.3389/fonc.2021.659048 PMC8019922

[59] Šteňo A, Buvala J, Toma D, Jezberová M, Šteňo J (2022) Navigated 3D-ultrasound power doppler and visualization of lenticulostriate arteries during resections of insular gliomas. Brain Spine 2:100873. 10.1016/j.bas.2022.100873 36248161 10.1016/j.bas.2022.100873 PMC9560660

[60] Šteňo A, Guissani C, Riva M (2016) Multimodal imaging in glioma surgery. In: Prada F, Solbiati L, Martegani A, DiMeco F (eds) Intraoperative ultrasound (IOUS) in neurosurgery—from standard B-mode to elastosonography. Springer, Cham Heidelberg New York Dordrecht London, pp 81–97

[61] Šteňo A, Hollý V, Mendel P, Šteňová V, Petričková Ľ, Timárová G, Jezberová M, Belan V, Rychlý B, Šurkala J, Šteňo J (2018) Navigated 3D-ultrasound versus conventional neuronavigation during awake resections of eloquent low-grade gliomas: a comparative study at a single institution. Acta Neurochir 160:331–342. 10.1007/s00701-017-3377-8 29150795 10.1007/s00701-017-3377-8

[62] Šteňo A, Jezberová M, Hollý V, Timárová G, Šteňo J (2016) Visualization of lenticulostriate arteries during insular low-grade glioma surgeries by navigated 3D ultrasound power doppler: technical note. J Neurosurg 125:1016–1023. 10.3171/2015.10.JNS151907 26848921 10.3171/2015.10.JNS151907

[63] Šteňo A, Karlík M, Mendel P, Čík M, Šteňo J (2012) Navigated three-dimensional intraoperative ultrasound-guided awake resection of low-grade glioma partially infiltrating optic radiation. Acta Neurochir 154:1255–1262. 10.1007/s00701-012-1357-6 22555551 10.1007/s00701-012-1357-6

[64] Sturiale CL, Scerrati A, Ricciardi L, Rustemi O, Auricchio AM, Norri N, Piazza A, Ranieri F, Benato A, Tomatis A, Albanese A, Mangiola A, Di Egidio V, Zotta DC, Farneti M, Marchese E, Raco A, Volpin L, Trevisi G (2023) Comparison between Intrasylvian and Intracerebral Hematoma Associated with ruptured Middle cerebral artery aneurysms: clinical implications, technical considerations, and outcome evaluation. World Neurosurg 173:e821–e829. 10.1016/j.wneu.2023.03.024 36906087 10.1016/j.wneu.2023.03.024

[65] Toyooka T, Otani N, Wada K, Tomiyama A, Takeuchi S, Fujii K, Kumagai K, Fujii T, Mori K (2018) Head-up display may facilitate safe keyhole surgery for cerebral aneurysm clipping. J Neurosurg 129(4):883–889. 10.3171/2017.5.JNS162692 29192858 10.3171/2017.5.JNS162692

[66] Uhl E, Zausinger S, Morhard D, Heigl T, Scheder B, Rachinger W, Schichor C, Tonn JC (2009) Intraoperative computed tomography with integrated navigation system in a multidisciplinary operating suite. Neurosurgery 64(5 Suppl 2):231–240. 10.1227/01.NEU.0000340785.51492.B5 19404103 10.1227/01.NEU.0000340785.51492.B5

[67] Unsgaard G, Ommedal S, Rygh OM, Lindseth F (2007) Operation of arteriovenous malformations assisted by stereoscopic navigation-controlled display of preoperative magnetic resonance angiography and intraoperative ultrasound angiography. Neurosurgery 61(1 Suppl):407–415. 10.1227/01.neu.0000279232.43202.82 18813150 10.1227/01.neu.0000279232.43202.82

[68] Unsgaard G, Rygh OM, Selbekk T, Müller TB, Kolstad F, Lindseth F, Hernes TA (2006) Intra-operative 3D ultrasound in neurosurgery. Acta Neurochir 148:235–253. 10.1007/s00701-005-0688-y 16362178 10.1007/s00701-005-0688-y

[69] Unsgård G, Rao V, Solheim O, Lindseth F (2016) Clinical experience with navigated 3D ultrasound angiography (power doppler) in microsurgical treatment of brain arteriovenous malformations. Acta Neurochir 158:875–883. 10.1007/s00701-016-2750-3 26993142 10.1007/s00701-016-2750-3 PMC4826661

[70] Woydt M, Greiner K, Perez J, Krone A, Roosen K (1997) Intraoperative color duplex sonography of basal arteries during aneurysm surgery. J Neuroimaging 7:203–207. 10.1111/jon199774203 9344000 10.1111/jon199774203

[71] Yang Y, Richard SA, Lan Z (2022) The impact of residual hematoma after evacuation on the outcomes of patients with ruptured intracranial aneurysms with intracerebral hematoma: a longitudinal single-center observational study. Med (Baltim) 101(36):e30129. 10.1097/MD.0000000000030129 10.1097/MD.0000000000030129 PMC10980503 36086761

[72] Zafar SF, Westover MB, Gaspard N, Gilmore EJ, Foreman BP, OʼConnor KL, Rosenthal ES (2016) Interrater agreement for consensus definitions of delayed ischemic events after aneurysmal subarachnoid hemorrhage. J Clin Neurophysiol 33:235–240. 10.1097/WNP.0000000000000276 27258447 10.1097/WNP.0000000000000276 PMC4894325

